# A Model for Implementing Psychotherapy for Individuals With Cognitive Impairments

**DOI:** 10.1080/07317115.2026.2699243

**Published:** 2026-07-15

**Authors:** Adrienne L. Johnson, Mary F. Wyman, Megan Gjertsen, Samantha Shapiro, Sheniqua Bouges, Jonathan Singer, Nathanial Chin

**Affiliations:** a Department of Medicine, University of Wisconsin School of Medicine and Public Health, Madison, Wisconsin, USA;; b Center for Tobacco Research and Intervention, University of Wisconsin, Madison, Wisconsin, USA;; c Geriatric Research, Education and Clinical Center (GRECC), W.S. Middleton Memorial Veterans Hospital, Madison, Wisconsin, USA;; d Department of Psychological Sciences, Texas Tech University, Lubbock, Texas, USA;; e Pharmacology and Neuroscience, Texas Tech University Health Sciences Center, Lubbock, Texas, USA;; f Garrison Institute on Aging, Texas Tech University Health Sciences Center, Lubbock, Texas, USA

**Keywords:** ADRD, MCI, psychotherapy

## Abstract

**Objectives:**

Individuals with mild cognitive impairment (MCI) and Alzheimer’s disease and Alzheimer’s disease–related dementias (AD/ADRD) are at greater risk for mental health concerns than those without cognitive impairments. We report the implementation of psychological services for patients with MCI and mild AD/ADRD within a large public health-care system in the United States.

**Methods:**

Preimplementation process included identifying a clinic champion. Referrals were initially provided by the clinic champion and then opened to the entire Memory Assessment Clinic with predetermined eligibility guidelines. Patients were seen on a short-term basis, using CBT- or ACT-based therapy. Initial sessions were conducted in person; follow-up sessions were in person and via telehealth.

**Results:**

Patients saw an average of a 2.2-point reduction in depression symptoms and a 2-point reduction in anxiety symptoms. Implementation barriers and facilitators are listed in the full-text of the article.

**Conclusions:**

This clinic implementation demonstrates the feasibility of evidence-based psychotherapy clinics within existing geriatric clinics. Future work should examine the impact of CBT and ACT to treat anxiety and depression in patients with these neurocognitive disorders. CLINICAL IMPLICATIONS: Patients with MCI and mild AD/ADRD show a clear interest in psychotherapy services, and initial efficacy data suggest there is a clinical benefit for mental health symptoms.

As the population ages, rates of cognitive impairment and Alzheimer’s disease and Alzheimer’s disease–related dementias (AD/ADRD) are rising rapidly in the United States, with over 14 million adults with mild cognitive impairment (MCI) and over 7 million with Alzheimer’s disease (Rajan et al., 2021). Patients with cognitive impairment are at increased risk for depression and anxiety symptoms compared with older adults in general. Older adults in general have depression rates estimated at 19%; whereas, depression rates are estimated at 32% in patients with MCI and 39% in patients with AD/ADRD (Jalali et al., 2024; Leung et al., 2021; Ma, 2020). Similarly, rates of anxiety in the general population of older adults are estimated at 19%, with rates as high as 31% in older adults with MCI and 39% in older adults with AD/ADRD, with higher rates among individuals in clinical or hospital settings (Jalali et al., 2024; Leung et al., 2021; Ma, 2020). This increased risk is especially relevant as it highlights the need for treatment among not just older adults in general but especially those with cognitive impairment. Anxiety and depression in people with MCI and AD/ADRD increases risk for cognitive and functional impairment, decreases quality of life, and increases caregiver stress (Cipriani et al., 2015; Seignourel et al., 2008). Moreover, research has shown that antidepressants are no more effective than placebos in treating depression among people with cognitive impairments, particularly when there is no depression prior to the neurocognitive diagnosis (Livingston et al., 2024). Another systematic review of people with ADRD/AD who do not have a major depressive disorder diagnosis showed that nonpharmacological interventions are more effective at reducing depressive symptoms in people with AD/ADRD than antidepressant medications (Watt et al., 2021). Similarly, there is mixed evidence on the effectiveness of antidepressants for anxiety in patients with dementia (Bingley et al., 2025). Moreover, a recent national cohort study in Sweden of patients with AD/ADRD found that current antidepressant use was associated with faster cognitive decline than nonuse (Mo et al., 2025).

Fortunately, multiple reviews and meta-analyses have found positive effects of psychotherapy on psychological distress in this patient population (Orgeta et al., 2022; Robinson & Moghaddam, 2022; Sukhawathanakul et al., 2021). Reviews covered a wide range of approaches but found the strongest effects among cognitive behavioral therapies (CBTs), with particular benefit on depression symptoms, and noting that anxiety symptoms were less commonly tracked (Orgeta et al., 2022; Robinson & Moghaddam, 2022). A 2021 systematic review examining different psychotherapeutic Interventions for patients with dementia found that problem-focused and meaning-based therapies had particularly positive impacts on psychological distress (Sukhawathanakul et al., 2021). Although two reviews found that mindfulness or third wave therapies did not significantly improve depression or anxiety symptoms, the reviews noted that this was due to the limited number of studies in this area and that more research is needed (Han, 2022; Orgeta et al., 2022).

Despite the growing evidence base for the effectiveness of these approaches for older adults with cognitive disorders, the availability of therapy for these patients remains severely limited (Cheston & Ivanecka, 2017; Committee on the Mental Health Workforce for Geriatric et al., 2012). This is problematic as the number of adults 65 and older with mental health or substance use disorders was 5.6–8 million in 2013; the number is expected to reach 10.1 to 14 million in the next 5 years (Committee on the Mental Health Workforce for Geriatric et al., 2012). Provider bias (e.g., providers feeling patients with cognitive impairment cannot engage/benefit in therapy) can also limit the availability of therapy for patients with cognitive impairments. For example, a 2018 study showed that primary care physicians’ perceptions of CBT impacted their willingness to refer their older patients for this treatment and a preference to treat patients themselves with medication and “watchful waiting” (Collins & Corna, 2018). Moreover, lack of confidence in one’s ability to provide high-quality therapy for patients with cognitive impairments as well as competing demands of health systems likely bias therapists’ willingness to work with these patients (Baker et al., 2022; Wyman et al., 2020). System barriers are particularly difficult to overcome, with some health-care systems ruling out psychothereapy services for any diagnosed cognitive impairment (Baker et al., 2022). In terms of patient interest, research shows that adults with psychiatric distress are three times more likely to prefer nonpharmacological interventions than pharmacological ones (McHugh et al., 2013). Thus, although patient interest is high and evidence-based treatments are available for patients with MCI and AD/ADRD, there remains a gap in access to nonpharmacological services. Feasible models for better integrating such services into health-care system structures are urgently needed.

This paper describes the implementation and early evaluation of an evidence-based therapy clinic, utilizing CBT (Beck, 2011) or acceptance commitment therapy (ACT) (Hayes et al., 1999, 2012) in the memory assessment clinic (MAC) of a large health-care center in the Midwestern United States. Using the organizing framework developed for the QUERI Implementation Roadmap (Kilbourne et al., 2019), we describe the preimplementation, implementation (including barriers and facilitators), and sustainment phases for this clinic and present preliminary data on patient population and psychiatric symptom outcomes of treatment. Finally, we offer recommendations for future directions in geriatric mental health care.

## Methods

The institutional review board at the University of Wisconsin approved the study (ID: 2024–1179). The consent requirement was waived by the ethics committee given the retrospective nature of this clinical implementation project.

### Description of clinic

The MAC is housed in a small outpatient clinic within the larger health system. This clinic includes geriatric and family medicine practices. From September 2020 to September 2021 there were 3,255 unique patients seen in this geriatric medicine clinic, with 25% of these patients having a diagnosis of AD/ADRD due to any etiology and an additional 14% of patients with MCI. Within this clinic there is a MAC that assesses patients over 65 years old for symptoms of AD/ADRD. Psychotherapeutic services for MAC patients were offered approximately half a day per week (four patients seen per week).

### Referral process

Initial referrals were solely from the clinical advocate. The initial pilot period allowed the psychologist, over time, to develop specific referral guidelines appropriate for this clinic. For example, test cases demonstrated that patients assessed by the referring provider had moderate or more severe AD/ADRD such that they were typically unable to retain information long enough to engage in conversations appropriately and find benefit in this specific therapy model. Formal eligibility criteria were developed prior to accepting referrals from other clinicians (geriatricians, nurse practitioners) in the MAC. To be eligible for psychotherapeutic treatment, patients had to have had a recent MCI or mild AD/ADRD diagnosis within the past year (for the majority of patients, this also meant they had a Functional Assessment Staging Tool [FAST; Reisberg et al., 1985] score at level 3 or 4), showed a lack of rapid forgetting during medical examination (as determined by the referring provider), had comorbid anxiety or depressive symptoms (via patient self-report or elevated Geriatric Depression Scale–Short Form (Sheikh & Yesavage, 1986) [GDS-15] or Geriatric Anxiety Scale 10-Item Version (Mueller et al., 2015) [GAS-10] scores, which are widely used with older adults with cognitive decline (Callow et al., 2020; Tam et al., 2023) and were already a standard assessment at the medical appointment with referring provider), and/or had difficulty coping with life as a result of the cognitive diagnosis. To inform providers of this new clinical opportunity, the clinical psychologist presented at a monthly clinical meeting of the geriatric department describing this service, differentiating the novel clinic from preexisting psychiatry and behavioral health services (which did not allow providers to see patients with any form of cognitive impairment in their medical record), reviewing eligibility criteria, and introducing the referral process. The clinic champion also facilitated referrals by informing nurse practitioners who worked with him of the service and encouraging referrals when appropriate.

Referrals were sent with a direct message within the clinic charting platform to the psychologist, linking to the most recent clinical note. The psychologist then contacted the potential patient to discuss the focus and structure of the clinic and set up an initial therapy appointment if appropriate. Referring providers informed patients of the scheduling process, including the potential for up to multiple months of time before the initial appointment could be scheduled and an overview of what to expect from the clinic (e.g., the clinic does not focus on couples therapy, it is short term in nature, it differs from behavioral health and psychiatric services already offered).

### Therapy overview

The psychologist is trained from an accredited American Psychological Association site and internship in CBT and ACT and has clinical and research experience adapting both therapy approaches for cognitively impaired individuals (Dindo et al., 2020; Evans-Hudnall et al., 2018). These therapeutic modalities (ACT and CBT) were chosen for this cognitively impaired older adult population based on their strong behavioral focus (Beck, 2011; Hayes et al., 1999) and documented success as interventions with patients with comorbid medical conditions (Cully et al., 2017; Dindo et al., 2017) including those with cognitive impairments (Dindo et al., 2020; Paukert et al., 2010). Sessions lasted approximately 45 minutes each and were all initially conducted in person to facilitate rapport-building and allow accurate evaluation. Thereafter, sessions were provided primarily in person with some patients receiving telehealth treatment, per patient preference. The vast majority of patients were seen together with their caregiver or surrogate decision-maker if agreed upon by the patient. If privacy was requested by the patient, this was honored and caregivers were asked to step out. If the patient did not have a caregiver they were seen independently, but this was the minority of patients. The initial session focused on reviewing the format of therapy, discussing limits of confidentiality and the psychologist’s role in the clinic, reviewing patient’s psychiatric history, determining the appropriateness of therapy (based on patient’s ability to follow conversation during session and elevated depression or anxiety symptoms via GDS-15 (Sheikh & Yesavage, 1986) or GAS-10 (Mueller et al., 2015) along with clinical psychiatric interview), providing a diagnosis for psychiatric symptoms, and determining the modality of therapy (ACT or CBT). Session two focused on identifying treatment goals, psychoeducation regarding cognitive diagnosis, and the introduction of a coping skill. The content of remaining sessions varied based on the therapy modality used and the presenting symptoms of the patient but typically included identification of problematic cognitions and behaviors, introduction of new core processes of therapy (e.g., cognitive defusion/acceptance/contact with present moment/self-as-a-context/values/committed action from ACT, cognitive restructuring/behavioral activation/systematic exposure/problem-solving/relaxation and stress reduction from CBT) and related coping skills, reframing of patient’s identity within the context of having a neurocognitive disorder, communication skills training for both the patient and their caregiver, assigning homework using learned skills, and evaluation of homework effectiveness and roadblocks. Patients met with the clinician approximately every other week via in-person and telehealth visits.

### Caregiver involvement in psychotherapy treatment

Consistent with recommendations for patients with cognitive impairments receiving therapy, caregivers were involved in therapy sessions with patient consent (Robinson & Moghaddam, 2022). More specifically, recent calls to action have stated there is a critical need to include caregivers more effectively in health-care overall and in the treatment of patients with AD/ADRD (i.e., dyadic inventions) (Fields et al., 2024; Vranceanu et al., 2025). Providing a dyadic AD/ADRD intervention supports both members of the dyad through shared communication, coping, and mutual support. This treatment included the caregivers as they attended sessions and practiced learned skills presented in session. They also were educated on strategies to assist the patient with homework completion between sessions. Lastly, to support relationship quality and daily functioning, communication training was targeted for both the patient and the caregiver.

### Measures

Patient records were reviewed for demographics (age and gender) and clinical outcomes including number of visits, psychiatric diagnoses, baseline and follow-up anxiety and depressive symptoms. Follow-up assessments occurred between the halfway point and the end of treatment (i.e., 2–7 sessions after therapy initiation). Clinical significance of change in anxiety and depression was based on remittance of symptoms from a “clinically elevated” range at baseline to “nonclinical/minimal” range at follow-up, per GDS-15 (Sheikh & Yesavage, 1986) or GAS-10 (Mueller et al., 2015) scoring.

### Analyses

The overall implementation process is described including preimplementation, implementation, and sustainment phases. Implementation facilitators and barriers are identified, along with key stakeholders throughout the implementation process.

For this pilot project, symptom scale scores were examined descriptively rather than statistically, due to the small sample size with complete data.

## Results

### Implementation outcomes

#### Preimplementation

This program was clinician initiated, rather than initiated from the health care system or in the context of wider programmatic goals. After observing the gap in available mental health care options for older adults who had been recently diagnosed with a cognitive disorder, the lead author and licensed clinical health psychologist (ALJ) proposed the clinical focus—psychotherapeutic treatment for patients with cognitive impairment—to a key stakeholder: a practicing geriatrician in the MAC, associate director of geriatric memory clinics, and senior author (NC). After getting support from the geriatrician, the psychologist met with the medical director of ambulatory geriatric services to address primary logistical considerations, including identifying clinical space in the same location as the geriatrician, obtaining training for and access to the electronic health record, and badging for identification and building access purposes. To collect data prior to implementation, the psychologist shadowed the geriatrician for approximately four days during one month. This included sitting in on visits, observing the multidisciplinary team consensus process for AD/ADRD and MCI diagnosis, and meeting key clinic-level stakeholders including clinic administrators (e.g., managers, schedulers, front desk staff). During this orientation period, the geriatrician became the primary clinical champion for the clinical program, offering to serve as the pilot referral source for the psychologist and assisting with development of the implementation plan.

#### Implementation

##### Barriers.

Barriers to implementation included lack of awareness of this new clinic among potential referring providers, incorrect use of referral procedures (i.e., referring ineligible patients, scheduling patients on the psychologist’s schedule without the psychologist talking to patients first), limited psychologist availability (i.e., having only 4 hours of clinical availability each week with wait lists from 3–5 months), and lack of awareness of their neurocognitive diagnosis of patients.

When referring providers became aware of this new clinic, there was an influx of referrals to the psychologist, resulting in a significant backlog of patients for the provider to contact. Subsequently, there were multiple instances of patients calling front desk staff or referring providers directing the front desk staff to put them on the psychologist’s schedule without her knowledge. Although this may be a common form of scheduling in many clinics, given the consistency of patient visits (i.e., seen every other week), scheduling a patient on the psychologist’s schedule without her knowledge resulted in delays in scheduling follow-up appointments with existing patients and at times, double-booking the psychologist. This was particularly true given the limited availability of the psychologist and lack of other providers giving similar care. In addition to scheduling difficulties, multiple patients who were initially referred to the psychologist did not know why they were seeing the psychologist and not interested in therapy. Similarly, some referrals did not meet eligibility criteria as they were too cognitively impaired (i.e., moderate to severe AD/ADRD) or did not have significant psychiatric distress (i.e., elevated depression or anxiety symptoms). A final barrier was the lack of patient awareness of their diagnosis. Although all appropriately referred patients had a diagnosis of MCI or mild AD/ADRD, some were not aware of this diagnosis or what it meant, resulting in confusion during their first meeting with the psychologist.

##### Facilitators.

Facilitators to implementation included identifying a clinical champion, clarification of eligibility criteria for patients, education of referring providers on eligibility criteria and referral procedures, development of a patient wait list, and including psychoeducation on patient diagnosis in the first therapy session.

Education-referring providers included presenting at monthly department meetings and direct feedback either in person or via electronic message to the referring provider about the appropriateness of referrals and clarification of the referral process. The psychologist also met with front desk and scheduling staff to clarify the referral process as this was different than other referrals within the health system, which helped address issues with patients calling preemptively and trying to schedule with the psychologist without her knowledge. The development of the patient wait list also enabled the psychologist to give quick feedback to the referring provider about patient interest and potential wait times and in addressing patient concerns about whether they needed to do more to see the psychologist. Moreover, this wait list helped ensure that the psychologist was not overscheduling her limited availability. Finally, prior to the initial appointment, the psychologist reviewed patient medical records to accurately identify their specific neurocognitive disease profile. After reviewing administrative tasks (e.g., therapy duration and scheduling, psychologist’s role in clinic, notes in electronic medical records) and discussing confidentiality of topics discussed in therapy as well as limits of confidentiality in the initial session, the psychologist made it standard practice to ask the patient what their understanding of their neurocognitive diagnosis was. This informed the psychologists of the patient’s level of understanding of their diagnosis and the associated impairments that may occur to help her provide psychoeducation on the diagnosis, its etiology, and the potential progression.

#### Sustainment

Once the clinic had been established, the psychologist continued to implement multiple facilitators identified in the implementation process to ensure the ongoing success of the clinic. This included reeducation of referring providers via departmental meetings and direct feedback. Additionally, following up with providers on the success of a referral in terms of scheduling closed the loop for providers and promoted ongoing future referrals consistent with eligibility criteria. Moreover, having the referring provider inform patients of the scheduling process (including placement on a wait list) and warn them of the wait time required prior to scheduling an initial appointment also facilitated sustainment, in that the psychologist did not spend excess time calling a long list of patients and patients were screened soon after referral and then determined whether they were willing to wait. The psychologist also met with front desk staff on bi-annual basis given the high turnover rate of administrative staff and that this clinic’s referral process was different than the standard referral process. Finally, since implementing the standard practice of asking the patient what their neurocognitive diagnosis was, this became a regular part of the first session along with a clinical intake of psychiatric history and current symptoms. Since initiation of this clinical program, the psychologist receives approximately 4 to 8 referrals per month with a standard wait list of 2 to 5 months.

### Clinical outcomes

#### Patients treated

A total of *N* = 88 patients were seen in the clinic starting from the launch of the clinic on September 10, 2020, to December 30, 2024. Fourteen patients were seen for one session only, due to therapy not being indicated after initial evaluation was completed. Reasons that therapy was not indicated included lack of psychiatric diagnosis outside of mild or major neurocognitive disorder, inability to comprehend and engage in conversation during initial evaluation session, and lack of patient interest in engaging in therapy.

Of the 74 therapy eligible patients, the majority self-identified as women and were on average 77 years old. Eligible participants were diagnosed with a variety of psychiatric conditions including adjustment disorders, major depressive disorder, generalized anxiety disorder (with and without a history of panic attacks), and depressive and anxiety disorders due to a known medical condition (their known neurological diagnosis).

Due to the clinical nature of this project, consistent reporting of standardized depression and anxiety scores was not prioritized over self-report of symptom improvement and clinical judgment. Only 26 therapy eligible patients had baseline and follow-up psychiatric symptoms noted in clinical documents. The total number of sessions (among people with follow-up data) ranged from 3 to 17 sessions. See Table 1 for demographic and clinical information of patients seen in the psychotherapy clinic.

### Psychiatric symptoms

On average, patients reported both baseline anxiety and depression symptoms in the “mild” ranges. Patients reported average follow-up anxiety and depression symptoms in the “within normal limits” or “minimal” ranges. Patients saw an average of a 2.2-point reduction in depression symptoms and a 2-point reduction in anxiety symptoms within two to seven sessions of initiating therapy.

## Discussion

This paper describes the implementation and brief evaluation of a novel clinic aimed at providing psychotherapy treatment to older adults recently diagnosed with MCI or mild AD/ADRD. We demonstrate the feasibility of offering evidence-based psychotherapy for older adults with cognitive disorders within an existing geriatric clinic setting, utilizing warm hand-offs from geriatric providers within the clinic to streamline the process and reduce barriers to treatment access for patients with cognitive disorders. Importantly, most of the patients who received treatment in the clinic were seen together with a caregiver at all sessions, which was found to be feasible. Our findings add to the growing literature on mental health service delivery for older adults with cognitive disorder (Cheston & Ivanecka, 2017).

Analysis of barriers and facilitators revealed the importance of a team-based approach and ongoing cross-disciplinary communication to implementation. Consistent with previous implementation studies (Bunce et al., 2020), working closely with a provider champion was a key strategy for reinforcing best practices to colleagues and effectively addressing other barriers to implementation. This champion also held a leadership role, solidifying organizational support for the new initiative, which was important for success (Moore et al., 2017). Consistent with previous work highlighting the role of staff education in improving dementia care (Mayrhofer et al., 2016), it was important for the treating psychologist to address the lack of understanding and knowledge about the brief therapy model, about the research evidence supporting the efficacy of psychotherapy and the mixed evidence on use of antidepressant medications for older adults with cognitive deficits. Moreover, consistent communication from the psychologist to all providers and administrative staff and clarifying appropriate referrals and addressing inaccurate information was crucial for facilitating uptake of the service and sustainment of the clinic model.

Using available clinical data, our initial evaluation of this brief therapy model demonstrated clinically significant reductions in depression and anxiety symptoms. This is consistent with previous reports of reductions in individuals with other neurological disorders (Soltaninejad et al., 2025). Especially in light of the brief follow-up period and the fact that some follow-up assessments were administered prior to the end of treatment, these findings are particularly promising. Symptom improvements are also consistent with findings from previous reviews of psychotherapy studies for adults with MCI and mild AD/ADRD (Orgeta et al., 2022; Robinson & Moghaddam, 2022; Sukhawathanakul et al., 2021). Other forms of psychotherapy have been tested with patients with cognitive impairments and comorbid anxiety and/or depressive symptoms (Carpenter et al., 2003; Craig et al., 2018; Kiosses et al., 2015; Kraus et al., 2008). However, to the best of the authors’ knowledge, this is the first study to utilize ACT and to use either ACT or CBT within a geriatric clinical setting specializing in dementia diagnosis. Although the diagnostic clinic served patients with a range of cognitive disorders, the brief treatment model and focus of these sessions did not support offering the program to patients with more-advanced levels of cognitive impairment. Previous efforts (Carpenter et al., 2003) have found success for psychotherapy in patients with moderate AD/ADRD, however, and future work should systematically examine the impact of CBT and ACT in the treatment of anxiety and depression in patients with MCI and mild AD/ADRD, using larger samples and standardized assessment procedures and should explore adaptations for those with moderate AD/ADRD.

There is a critical need to improve the scope and depth of services offered to older adults and their families after a diagnosis of dementia or MCI (Dooley et al., 2025). Although collaborative care models using psychotherapy delivered to older adults within a medical practice have been widely successful in increasing access and treating symptoms (Arean et al., 2008), such integrative approaches for older adults with dementia are less common, despite the high rate of mental health concerns in this population (Jalali et al., 2024; Leung et al., 2021; Ma, 2020). Increasing accessibility of services is associated with higher utilization of psychotherapy among older adults (Nurit et al., 2016), and this implementation demonstrates the feasibility of offering evidence-based psychotherapy for older adults with cognitive deficits within existing geriatric medical clinics, particularly clinics providing diagnostic services for cognitive impairment. We also affirm the feasibility of including caregivers in the psychotherapy treatment sessions—consistent with past psychotherapy trials (Spector et al., 2015)—who may play an especially critical role in increasing mental health utilization for older adults, especially those with dementia (Wyman et al., 2024). Our study can inform future efforts toward increasing access to mental health care for persons with cognitive deficits and changing the ecosystem of dementia care (Bonner et al., 2023).

## Limitations

There are multiple limitations of this work to consider. First, due to the clinical nature of this project, consistent reporting of standardized depression and anxiety scores was not prioritized and therefore the number of patients with baseline and follow-up data was limited and the follow-up period was inconsistent. Future studies should systematically assess pre-and posttreatment anxiety and depression scores in patients and assess caregiver perspective of impact of treatment on the patient’s emotional health and the caregiver’s own (Fields et al., 2024). Second, this study did not split analyses by type of therapy offered (CBT and ACT). Future work should independently examine the effect of CBT versus ACT to determine the most effective psychotherapeutic treatment in this population. Third, the psychologist initiated this service and came with required experience necessary to treat these patients. Workforce shortages among geriatrics-trained mental health care providers have been well documented (Lester et al., 2020; Moye et al., 2019). Future work in this area should examine models for disseminating skills and adaptations used to provide evidence-based psychotherapies to cognitively impaired individuals.

## Conclusion

This clinic implementation demonstrates the feasibility of the operation of evidence-based psychotherapy clinics, with relatively little preimplementation process, within existing geriatric clinics, particularly those providing diagnostic services for cognitive impairment. Older adults with cognitive impairments treated with CBT- or ACT-based therapy saw significant improvements in their anxiety and depressive symptoms.

## Figures and Tables

**Table 1.** Patient demographics and clinical outcomes.

Total patients seen for initial session	88
Therapy eligible patients	74
Median number of sessions of eligible patients	4
Gender of eligible patients–N (%) female	52 (70.3%)
Age of eligible patients in years– M(SD)	77.4 (5.9)
Psychiatric diagnoses of eligible patients	Adjustment disorder (mixed, with depressive-like episode, with anxiety), major depressive disorder, generalized anxiety disorder, anxiety disorder due to medical condition, depressive disorder due to medical condition
Baseline Geriatric Anxiety Scale 10-Item* - M(SD)	7.7 (3.4)
Follow Up Geriatric Anxiety Scale 10-Item* - M(SD)	5.7 (2.9)
Baseline Geriatric Depression Scale – Short Form * - M(SD)	5.1 (2.7)
Follow Up Geriatric Depression Scale – Short Form * - M(SD)	2.9 (1.9)

* *N* = 26 individuals who had baseline *and* follow up scores for either anxiety and/or depression.

## Data Availability

The participants of this study did not give written consent for their data to be shared publicly. Due to the sensitive nature of the research, supporting data are not available.

## Clinical implications

- This clinic implementation demonstrates that it is feasible to implement evidence-based psychotherapy clinics at existing geriatric clinics.
- Patients with MCI and mild AD/ADRD show a clear interest in psychotherapy services, and initial efficacy data suggest that there is a clinical benefit for mental health symptoms.
- Facilitators to implementation include identification of a clinic champion, clarification of clinic eligibility, education and reeducation of referring providers on the referral process, and creation of a wait list.
